# The respiratory syncytial virus nucleoprotein–RNA complex forms a left-handed helical nucleocapsid

**DOI:** 10.1099/vir.0.053025-0

**Published:** 2013-08

**Authors:** Saskia E. Bakker, Stéphane Duquerroy, Marie Galloux, Colin Loney, Edward Conner, Jean-François Eléouët, Félix A. Rey, David Bhella

**Affiliations:** 1 Medical Research Council – University of Glasgow Centre for Virus Research, Church Street, Glasgow, G11 5JR, UK; 2 Institut Pasteur, Unité de Virologie Structurale, Département de Virologie and CNRS Unité de Recherche Associée (URA) 3015, 25 Rue du Dr Roux, 75724 Paris Cedex 15, France; 3 INRA, Unité de Virologie et Immunologie Moléculaires, Domaine du Vilvert, 78350 Jouy-en-Josas, France; 4 Université Paris Sud, 91405 Orsay, France

## Abstract

Respiratory syncytial virus (RSV) is an important human pathogen. Its nucleocapsid (NC), which comprises the negative sense RNA viral genome coated by the viral nucleoprotein N, is a critical assembly that serves as template for both mRNA synthesis and genome replication. We have previously described the X-ray structure of an NC-like structure: a decameric ring formed of N-RNA that mimics one turn of the helical NC. In the absence of experimental data we had hypothesized that the NC helix would be right-handed, as the N–N contacts in the ring appeared to more easily adapt to that conformation. We now unambiguously show that the RSV NC is a left-handed helix. We further show that the contacts in the ring can be distorted to maintain key N–N-protein interactions in a left-handed helix, and discuss the implications of the resulting atomic model of the helical NC for viral replication and transcription.

Respiratory syncytial virus (RSV) causes lower respiratory tract infections in infants and the elderly. Like other Paramyxoviridae, RSV has a non-segmented negative sense RNA genome that is coated by the viral nucleoprotein N to form the nucleocapsid (NC), which serves as the template for RNA synthesis by the viral RNA-dependent RNA polymerase. The NC is a flexible helix, with variable pitches and numbers of N-proteins per turn of the helix. This variability has hampered the structural characterization of the RSV NC (Bhella *et al.*, 2002, 2004). We have previously described the X-ray structure of an RSV NC-like complex, a decameric ring of N-protein in complex with RNA (Tawar *et al.*, 2009). The structure showed that the N-protein has a core region composed of N- and C-terminal domains (the ‘N-core’), with the RNA bound in a groove between them. In addition, an N-terminal and a C-terminal extension, called N-arm (residues 1–28) and C-arm (residues 360–375), invade the 5′ and the 3′ adjacent subunits in the RNA-bound ring, respectively. This results in a stable yet flexible interaction between subunits, with only a few direct contacts between adjacent N-cores. In particular, an α-helix in the N-arm inserted into the hydrophobic core of the neighbouring 5′ N-protein provides a major stabilizing interaction. The structure suggests multiple possible alternative conformations of the linker to the N- and C-arms in a helix, providing an explanation to the observed flexibility of the RSV NC. As the overall helical diameter of the NC is similar to that of the decameric ring, we generated a model for the NC helix with similar N–N contacts as those observed in the ring. In the absence of experimental data confirming the handedness, we proposed a right-handed helix, which appeared to allow better preservation of the interactions between subunits (Tawar *et al.*, 2009).

Here we set out to determine unequivocally the handedness of the helical RSV NC by electron tomography of purified NC-like ribonucleoprotein complexes (RNPs). To provide a control for our methods, we also analysed tomograms of measles virus (MeV) RNPs, which are known to be left-handed helices (Schoehn *et al.*, 2004). RNPs were prepared by recombinant expression of the relevant N-protein in insect cells as previously described (Bhella *et al.*, 2002). Tomography of purified RNPs embedded in negative stain was performed in a JEOL 2200 FSC TEM (for a full description of the methods, see the supplementary material, available in *JGV* Online). We found that both preparations formed helical and ring-like structures, although the MeV preparation appeared to contain more regular helices than that of RSV. In agreement with our previous studies, we found that the MeV and RSV helices had a diameter of 21 nm and 15 nm, respectively.

We recorded ten tilt-series of images for RSV RNPs and seven for MeV RNPs, and processed them to calculate tomograms using IMOD (Kremer *et al.*, 1996). To improve the contrast in these three-dimensional reconstructions, they were denoised using the non-linear anisotropic diffusion method (Fig. 1a) (Frangakis & Hegerl, 2001). These reconstructions revealed apparently left-handed helical organizations in both preparations, although noise and limitations on resolution in the *z*-axis brought about by the missing wedge introduced some uncertainty (Fig. S1). We therefore proceeded to calculate subtomogram averages using Dynamo (Castaño-Díez *et al.*, 2012). The resulting reconstructions showed that both MeV and RSV NC-like helical RNPs are left-handed, with a pitch of 52 Å and 68 Å, respectively (Figs 1b and S1). The resolution was too low to determine the twist (number of subunits per helical turn), possibly due to the inherent flexibility of paramyxovirus NCs. Helical symmetry was therefore imposed on the structures to give a clearer view of the left-handed conformation (Fig. 1c).

**Fig. 1.**
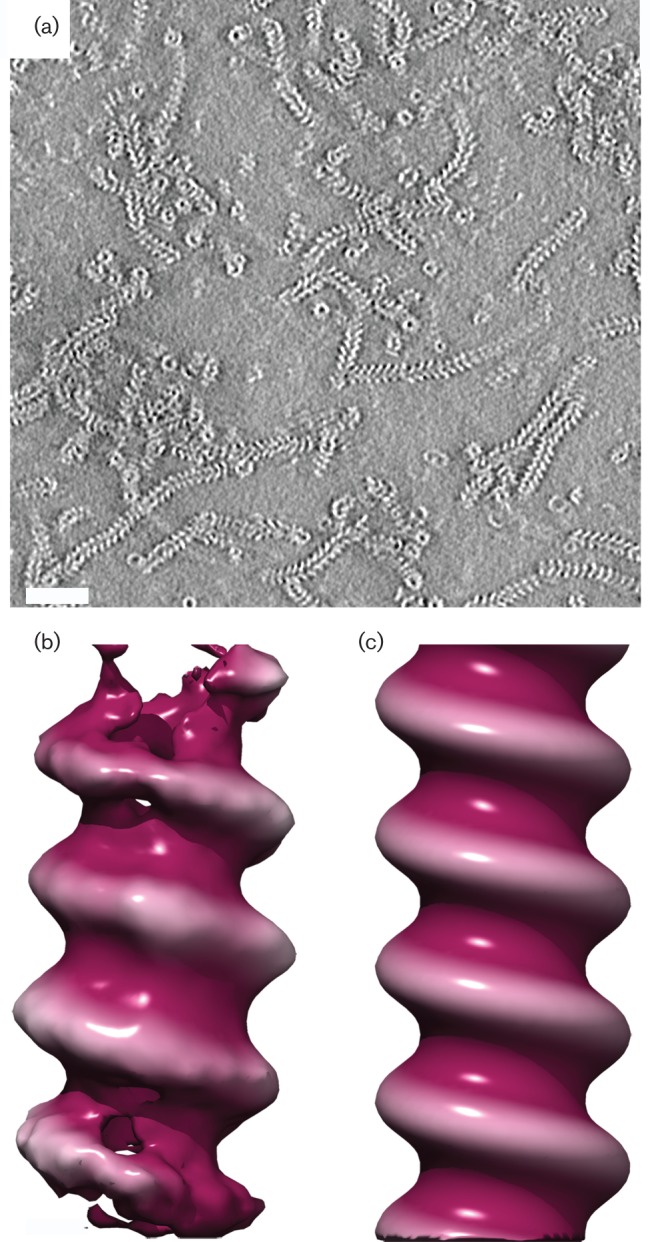
Tomography of RSV nucleocapsid. (a) A central section through a denoised tomogram of RSV NC-like RNPs reveals the presence of both rings and short helices. (b) Isosurfaced views of subtomogram averages for RSV nucleocapsid-like RNP reveal that these assemblies are left-handed helices. As the reconstructions did not have sufficient resolution to determine the twist (number of subunits per helical turn), symmetry was imposed. (c) Symmetrized RNP from RSV N-RNA.

Based on these experimental observations, and considering that the ring mimics one turn of the helical NC, we generated an atomic model for a left-handed RSV NC maintaining the contacts observed in the crystal structure using a procedure previously described (Tawar *et al.*, 2009) (Fig. S2). A composite subunit was assembled including the coordinates of the N-core and seven RNA nucleotides most closely bound to it, together with the N- and C-arms of its immediate neighbours on either side. This ensemble was replaced as a single rigid body in the helix to preserve the intricate contacts made between the component parts. Each composite subunit was then rotated about the helical axis (instead of the ring axis) by an angle close to 2π/10 (the rotation is exactly 2π/10 in the ring) and translated along the helix axis by a distance defined by the measured helical pitch (i.e. 68 Å/10). To best preserve the contacts in the ring, a rotation of the composite subunit about a radial axis (perpendicular to the helix axis) prior to the application of the helical operator is necessary. This rotation, the angle of which depends on the helical pitch, allows the adjacent subunits to face each other in a manner closely resembling that seen in the ring, after the application of the helical transformation. To achieve this, the simplest approach is to apply the rotation around the centre-of-mass (COM) of the subunit, and then to apply the helical operator. This operator has a translation defined by the pitch of the helix, and a rotation angle such that the COM to COM distance between adjacent subunits remains the same as in the ring. This rigid-body operation, however, results in breaking some of the contacts observed in the ring, and also introduces certain clashes, in particular to residues located away from the COM. Although the close contacts of the N-core with its neighbours are scarce, there are a few key contacts between N-core atoms, in particular the interactions of the Arg234 side-chain, which links three consecutive subunits (Fig. 2b, c). Thus, Arg 234 from subunit N_−1_ makes a bidentate salt bridge with the Asp221 side-chain of N_0_ whilst simultaneously hydrogen bonding the side-chain of Tyr23 in the N-arm of the second neighbour along the chain (N_+1_) (the subunits are numbered in the 5′ to 3′ direction of the RNA along the ring). Modelling the helix using the COM of the C_α_ of Asp221 and Tyr23 from protomers on each side of the subunit as the centre of rotation about the radial axis, rather than rotating about the COM of the whole subunit, resulted in a model for the helical NC with minimal clashes that preserved the inter-subunit hydrogen bonding described above (Fig. 2b, c). Elimination of minor clashes, reconnection of the arms and the RNA chain after the applied rigid-body movement to generate a continuous NC helix was done by energy minimization using REFMAC (Murshudov *et al.*, 2011). Superposing the N-core of one subunit in the ring with its counterpart in the final helical NC resulted in a root mean square deviation (r.m.s.d.) of 3.9 Å between C_α_ atoms in the immediate corresponding neighbour, with the largest displacement of about 8 Å (Fig. 2b, Table S1). Small changes in the parameters used to generate the atomic model compared to those used previously (Tawar *et al.*, 2009) have thus resulted in a left-handed helix in which key inter-subunit interactions were maintained, whereas previously the left-handed model had been rejected because inter-subunit interactions appeared unfavourable. As the twist of the RSV NC was not defined by our subtomogram averaging experiment, we are not able to discuss interactions between N-subunits in successive turns. The revised model is a repetition of exactly the same contacts along the helical NC, although in reality each N-subunit is likely to adopt slightly different orientations with respect to its neighbours through flexibility about the hinge between N-core and the arms. Importantly, the intricate hydrogen-bonding pattern of Arg234 was preserved. To test the result of breaking these interactions, we mutated Arg234 to alanine and tested the mutant N-protein’s activity with the polymerase using an HRSV plasmid based minigenome system as described previously (Fix *et al.*, 2011; Tran *et al.*, 2009). Briefly, the dicistronic subgenomic replicon pM/Luc, was co-transfected into BSRT7/5 cells expressing T7 RNA polymerase, together with plasmids pN, pP, pL and pM2-1, resulting in replication and transcription of the minigenome. Hence, production of the Luc protein was dependent on these processes. The activity of the R234A mutant was reduced to 68±8 % compared to the wt N, indicating that the Arg234 interactions are important but not essential (Fig. 2d).

**Fig. 2.**
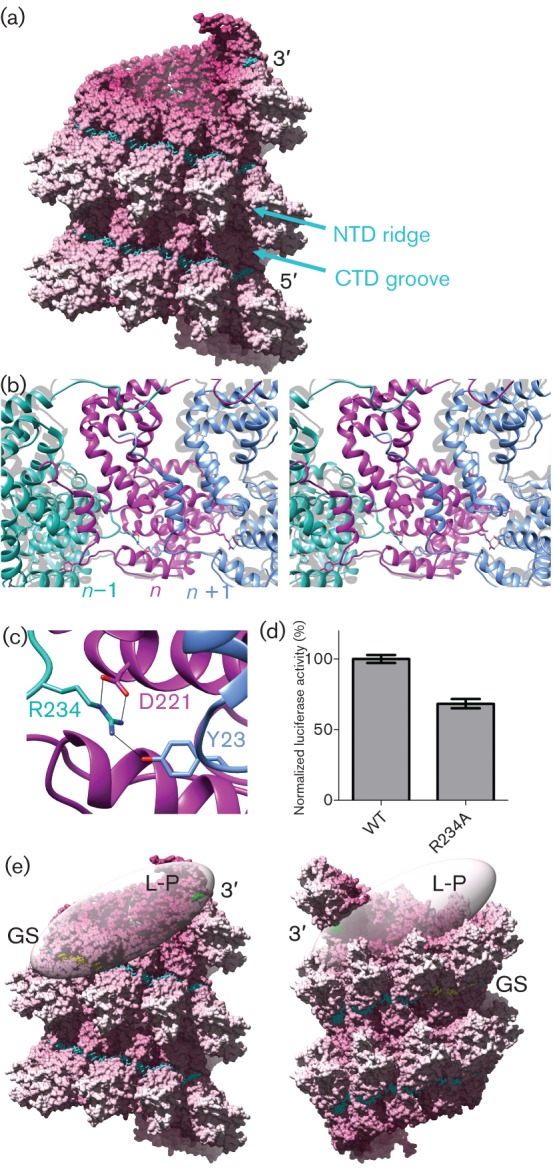
Modelling the RSV nucleocapsid. (a) The left-handed helix obtained by maximizing the preservation of key N–N interactions observed in the ring. A surface rendering of the helix is shown in which the RNA is represented by cyan sticks. (b) Stereo view of the N–N interactions in the modelled helix. The N-terminal arm of subunit *n*+1 (blue) binds in the core of subunit *n* (magenta). The ribbon structure for three subunits from the ring was aligned to subunit *n* and is shown in grey. (c) The inter-subunit interactions are mediated by hydrogen bonds between residue Asp221 of subunit *n*, Tyr23 of subunit *n*+1, and Arg234 of subunit *n−*1 (coloured as in panel b). (d) Evaluation of the effect of R234A mutation on viral RNA synthesis using an RSV based minigenome assay revealed that the mutant N-protein reduced polymerase activity to 68±8 % of the WT level. (e) Implications of the left-hand versus right-handed helical NC for transcription initiation. The left-handed and right-handed NC helix models are shown in surface representation, with the RNA in cyan sticks, except for the nucleotides corresponding to the leader sequence at the extreme 3′ end of the genome and the first gene-start element, shown in green and yellow, respectively. The two domains of the N-protein, NTD and CTD, are such that adjacent CTDs form a continuous groove along the helix, and the NTDs form a helical ridge, labelled in the figure. In the left-handed NC, the bulky NTD projects away from the polymerase complex, allowing simultaneous access to both sequence elements, whereas in the right-handed NC helix, it would project toward the polymerase, interfering with access to the gene-start element.

Our tomographic analysis of recombinant NCs provides firm experimental evidence to revise our previous interpretation (Fig. S3). We have demonstrated that RSV has a left-handed NC, a feature that is in common with other Mononegavirales (Bharat *et al.*, 2011; Egelman *et al.*, 1989; Ge *et al.*, 2010; Schoehn *et al.*, 2004). The most important implication of this finding is that the 3′ end of the RSV genome is located at the pointed end of the NC, and not at the barbed end (Fig. 2a). This means a polymerase bound at the 3′ end of the viral genome might also have access to the RNA in the subsequent helix turn, which contains a gene-start (GS) element (Fig. 2e). The polymerase could then detach from the 3′ end and glide along the ‘CTD groove’ during transcription. In a right-handed helix, a polymerase complex interacting with the 3′ nucleotides would not have such ready access to the GS element and would require elongation to take place across the ‘NTD ridge’. Thus the polymerase would have to bind at the 3′ site and then travel along the genome to the GS signal. The geometrical organization of the transcription start site therefore appears to be more favourable in a left-handed helix.

Our atomic model for the RSV helical NC, along with the key polar interactions described, has the potential to inform further mutagenesis studies of N residues to establish which play important roles during RSV replication and transcription, thereby providing further targets for antiviral research.

## Data deposition

The subtomogram averages have been deposited in the EMDB, accession codes emd2369 (RSV) and emd2370 (MeV). The atomic model of the helix has been deposited in the PDB, accession code 4bkk.
